# Ursolic Acid Attenuates Diabetic Mesangial Cell Injury through the Up-Regulation of Autophagy via miRNA-21/PTEN/Akt/mTOR Suppression

**DOI:** 10.1371/journal.pone.0117400

**Published:** 2015-02-17

**Authors:** Xinxing Lu, Qiuling Fan, Li Xu, Lin Li, Yuan Yue, Yanyan Xu, Yan Su, Dongcheng Zhang, Lining Wang

**Affiliations:** 1 Department of Nephrology, the First Hospital of China Medical University, Shenyang, 110001, China; 2 Department of nephrology, the People’s Hospital of Liaoning Province, Shenyang, 110001, China; University of Alabama at Birmingham, UNITED STATES

## Abstract

**Objective:**

To investigate the effect of ursolic acid on autophagy mediated through the miRNA-21-targeted phosphoinositide 3 kinase (PI3K)/protein kinase B (Akt)/mammalian target of rapamycin (mTOR) pathway in rat mesangial cells cultured under high glucose (HG) conditions.

**Methods:**

Rat glomerular mesangial cells were cultured under normal glucose, HG, HG with the PI3K inhibitor LY294002 or HG with ursolic acid conditions. Cell proliferation and hypertrophy were assayed using an MTT assay and the ratio of total protein to cell number, respectively. The miRNA-21 expression was detected using RT-qPCR. The expression of phosphatase and tensin homolog (PTEN)/AKT/mTOR signaling signatures, autophagy-associated protein and collagen I was detected by western blotting and RT-qPCR. Autophagosomes were observed using electron microscopy.

**Results:**

Compared with mesangial cells cultured under normal glucose conditions, the cells exposed to HG showed up-regulated miRNA-21 expression, down-regulated PTEN protein and mRNA expression, up-regulated p85PI3K, pAkt, pmTOR, p62/SQSTMI, and collagen I expression and down-regulated LC3II expression. Ursolic acid and LY294002 inhibited HG-induced mesangial cell hypertrophy and proliferation, down-regulated p85PI3K, pAkt, pmTOR, p62/SQSTMI, and collagen I expression and up-regulated LC3II expression. However, LY294002 did not affect the expression of miRNA-21 and PTEN. Ursolic acid down-regulated miRNA-21 expression and up-regulated PTEN protein and mRNA expression.

**Conclusions:**

Ursolic acid inhibits the glucose-induced up-regulation of mesangial cell miRNA-21 expression, up-regulates PTEN expression, inhibits the activation of PI3K/Akt/mTOR signaling pathway, and enhances autophagy to reduce the accumulation of the extracellular matrix and ameliorate cell hypertrophy and proliferation.

## Introduction

Diabetic nephropathy (DN) is one of the major causes of end-stage renal disease, and the incidence of DN is increasing worldwide. However, the pathogenesis of this disease remains unclear, and there is currently no effective drug to reverse the resulting renal damage. Thus, there is an urgent need for a new therapeutic method to block DN progression.

Autophagy is a lysosome-dependent bulk degradation process that is highly conserved from yeasts to mammals. Autophagy is involved in the clearance of long-lived proteins, damaged and excess organelles, and intracellular pathogens to maintain the cellular dynamic balance and integrity of cells. LC3II has recently been suggested as a marker for autophagosome formation, and p62/SQSTMI has been implicated as a marker for autophagolysosome degradation [1–3]. In DN, the need for cellular protective function through autophagy increases, reflecting not only a decrease in autophagocytic capacity but also a high level of cellular stress and the generation of excess metabolic products [4]. Phosphoinositide 3 kinase (PI3K)/protein kinase B (Akt)/mammalian target of rapamycin (mTOR) signaling is a classic negative regulatory pathway for autophagy. PI3K is an upstream regulatory factor in AKT activation. Activated AKT phosphorylates the downstream mTOR, which plays a central role in cell hypertrophy, growth, and survival and protein synthesis [5]. Phosphatase and tensin homolog (PTEN) activation inhibits AKT/mTOR signaling, and current studies have confirmed that microRNA (miRNA)-21 targets PTEN to activate the AKT/mTOR pathway, which plays an important role in the key pathological damage observed in DN [6,7].

Ursolic acid (UA) is a pentacyclic triterpenoid compound used in Chinese herbal medicine. UA exhibits anti-tumor, anti-angiogenesis, liver protective and lipid-lowering effects, inhibits reactive oxygen species (ROS) formation, and has few toxic side effects [8–15]. Recent studies have reported that the anti-oxidant functions of UA inhibit lipid peroxidation in a gentamicin-induced renal injury rat model, thus exerting an additional renal protective function [16]. A previous study demonstrated that UA inhibits cell proliferation and induces apoptosis through the inhibition of miRNA-21 expression in the U251 glioblastoma cell line [17]. This study was the only report on the regulation of miRNA expression through UA. However, whether UA inhibits miRNA-21 expression in mesangial cells has not been reported. The aim of the present study was to investigate whether UA inhibits the up-regulation of miRNA-21 expression in mesangial cells under high-glucose (HG) conditions, thus up-regulating the expression of the target gene PTEN to inhibit the activation of the PI3K/Akt/mTOR signaling pathway and induce autophagy, thereby decreasing the accumulation of the extracellular matrix and exerting a renal protective effect.

## Materials and Methods

### 1. Cell culture and grouping

The rat mesangial cell line HBZY-1 was purchased from the China Center for Type Culture Collection and cultured in low-glucose Dulbecco’s modified Eagle’s medium (DMEM; Hyclone,USA) containing 10% fetal bovine serum (FBS; Hyclone,USA), 100 U/ml penicillin, and 100 U/ml streptomycin. The cells were routinely cultured at 37°C and 5% CO_2_ with saturated humidity. The cells were digested and passaged when the cell confluence was greater than 85%. At 24 h after passaging and cell attachment, the cells were divided into the following groups: A, the normal-glucose group (NG), which received 5.5 mmol/L glucose (Sigma); B, the hypertonic control group (MA), which received 5.5 mmol/L glucose+19.5 mmol/L mannitol (Sigma); C, the high-glucose group (HG), which received 25 mmol/L glucose; D, the LY294002 intervention group (LY294002), which received 25 mmol/L glucose+5 μmol/L LY294002 (CST; Sigma); and E, the UA intervention group (UA), which received 25 mmol/L glucose+2.5 μmol/L UA (Sigma).

### 2. Detection of cell survival and proliferation capacity using an MTT assay

Mesangial cells were plated into 96-well plates at a density of 3×10^3^ cells/well. After attachment for 24 h, the culture medium was replaced according to their grouping, and the cells were cultured for an additional 24, 48, or 72 h. The supernatants were discarded, and 200 μL of culture medium containing 0.5% 3-(4,5-dimethylthiazol-2-yl)-2,5-diphenyltetrazolium bromide (MTT; Sigma) was added to each well. After culturing for another 4 h, the culture medium was carefully removed, and 150 μL of dimethyl sulfoxide (DMSO; Sigma) was added to each well. The cells were placed in an incubator and vortexed at a low speed for 10 min to fully dissolve the crystals. The optical density (OD) of each well at 490 nm was measured using a microplate reader to calculate the cell proliferation using the following formulas: cell viability = (OD(Experimental Group)/OD(Control Group)) × 100%,proliferation rate = OD(Experimental Group)-OD(Control Group)/ OD(Control Group), and inhibition rate = OD(Control Group)-OD(Experimental Group)/ OD(Control Group).

### 3. Determination of cell hypertrophy as the amount of total protein/cell number

Mesangial cells were plated into 6-well plates and cultured in serum-free culture medium containing a normal glucose concentration for 24 h to synchronize the cells. Subsequently, the cells were cultured with medium from each group for 24, 48, and 72 h. The cells were digested with trypsin, and a small portion of the intact living cells was stained with trypan blue and counted using a hemocytometer. The other cells were lysed in radioimmunoprecipitation assay (RIPA) buffer, and the total protein concentration was measured using the bicinchoninic acid (BCA) method. The total protein/cell number (micrograms of protein/10^4^ cells) was calculated.

### 4. Real-time PCR detection

The total RNA from the cells in each group was extracted and transcribed into cDNA using random primers and mouse mammary tumor virus (MMTV) reverse transcriptase. The reverse-transcribed cDNA was used as a template for polymerase chain reaction (PCR) amplification using Taq DNA polymerase. The primer sequences used in the present study are listed in Table 1. The following reaction conditions were used: pre-denaturation at 94°C for 5 min, followed by 40 cycles of denaturation at 95°C for 10 s, annealing at 60°C for 30 s, and extension at 72°C for 30 s. Glyceraldehyde 3-phosphate dehydrogenase (GAPDH) was used as an internal control. The relative mRNA expression was quantified through a comparison of the cycle threshold (Ct) values. The experimental data were processed using the 2^-ΔΔCt^ method: ΔΔCt = (Ct target-Ct internal control) experiment group-(Ct target-Ct internal control) normal control group. Each experimental group was repeated 3 times.

**Table 1:** Primers used for quantitative real-time RT-PCR.

**Gene**	**Primer sequence5'-3'**	**Product size (bp)**
pre-miRNA-21	5'-TGTACCACCTTGTCGG-3'	63
	5'- TGCTGTTGCCATGAGAT-3'	
PTEN	5'-CCATAACCCACCACAG-3'	119
	5'-CAGTCCGTCCTTTCC-3'	
Collagen I	5'-AAACGGGAGGGCGAGTG-3'	238
	5'-CATAGGACATCTGGGAAGCAA-3'	
p62/SQSTMI	5'-TCGGAAGTCAGCAAACCT-3'	152
	5'-AAATGCGTCCAGTCGTCA-3'	
GAPDH	5'-CGTATCGGACGCCTGGTT-3'	124
	5'-CGTGGGTAGAGTCATACTGGAAC-3'	

### 5. Western blotting

Total protein was extracted from the cells in each group using pre-cooled RIPA lysis buffer containing protease inhibitors. Protein concentrations were determined using the Micro BCA Protein Kit. Approximately 30–50 μg of total protein was separated using sodium dodecyl sulfate polyacrylamide gel electrophoresis (SDS-PAGE). Subsequently, the proteins were transferred onto a nitrocellulose membrane. The membrane was blocked in 3% bovine serum albumin (BSA) at room temperature for 1 h, followed by incubation with different primary antibodies for 4 h with slow shaking and additional incubation at 4°C overnight. The primary antibodies included the following: anti-PTEN (Santa Cruz, 1:300 dilution); anti-p85 PI3K (CST, 1:800 dilution); anti-pAKT (CST, 1:800 dilution); anti-tAKT (CST, 1:800 dilution); anti-pmTOR (CST, 1:500 dilution); anti-type I collagen (Proteintech, 1:500 dilution); anti-LC3II (CST, 1:500 dilution); and anti-p62/SQSTMI protein (CST, 1:500 dilution). GAPDH (Proteintech, 1:2000 dilution) was used as an internal control. The membrane was washed 3 times with Tris-buffered saline containing Tween-20 (TBST) for 10 min and subsequently incubated with secondary antibody (Proteintech, 1:20,000 dilution) at room temperature for 1 h. After washing 3 times with TBST for 10 min, the membranes were developed using an enhanced chemiluminescence (ECL) solution and scanned. Semi-quantitative analysis was performed using ImageJ image analysis software.

### 6. Transmission electron microscopy

The cells from each group were digested after 48 h of culture, followed by centrifugation, and the floating cells were collected. The cells were washed twice with cold PBS and fixed in 5% glutaraldehyde. Subsequently, the cells were conventionally dehydrated, embedded, sectioned, and stained, and the formation of autophagosomes was observed using transmission electron microscopy. The number of intracellular autophagosomes in every ten fields was counted.

### 7. Statistical analyses

The experiments were repeated 3 times. The quantitative data are presented as x±s. The Statistical Package for Social Science (SPSS) 17.0 software was used for analysis. Comparisons among multiple groups were performed using one-way analysis of variance (ANOVA). Pair-wise comparisons were performed using the t test. P<0.05 indicated that the observed difference was significant.

## Results

### 1. Inhibition of HG-induced abnormal hypertrophy and mesangial cell proliferation through UA

**Fig 1:**
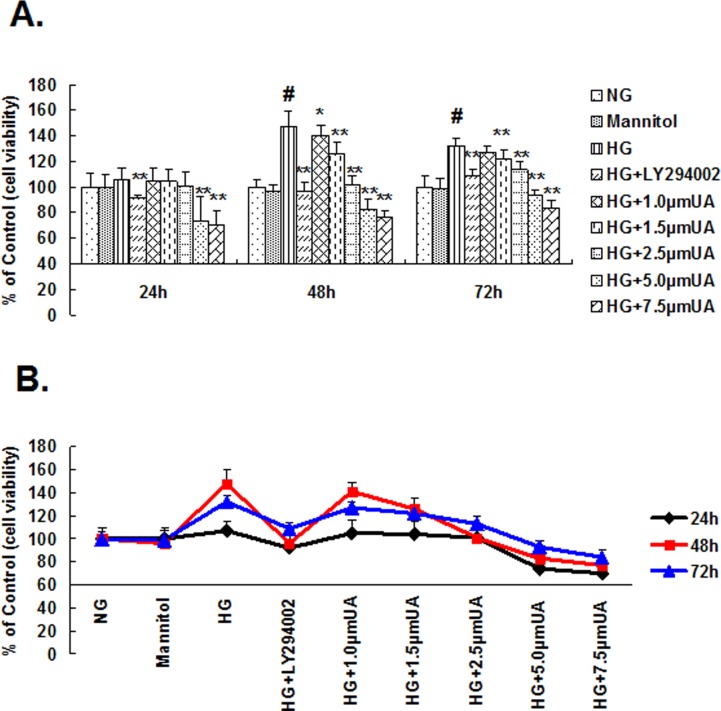
Ursolic acid inhibited HG-induced rat mesangial cell proliferation. (A and B) Mesangial cells were cultured in normal glucose and HG, HG with LY294002 or HG with ursolic acid (1.0, 1.5, 2.5, 5.0, and 7.5 μm) for 24, 48 and 72 h. Cell proliferation was detected using MTT. #, P<0.01 vs. NG; *, P<0.05 vs. HG; **, P<0.01 vs. HG.

**Fig 2:**
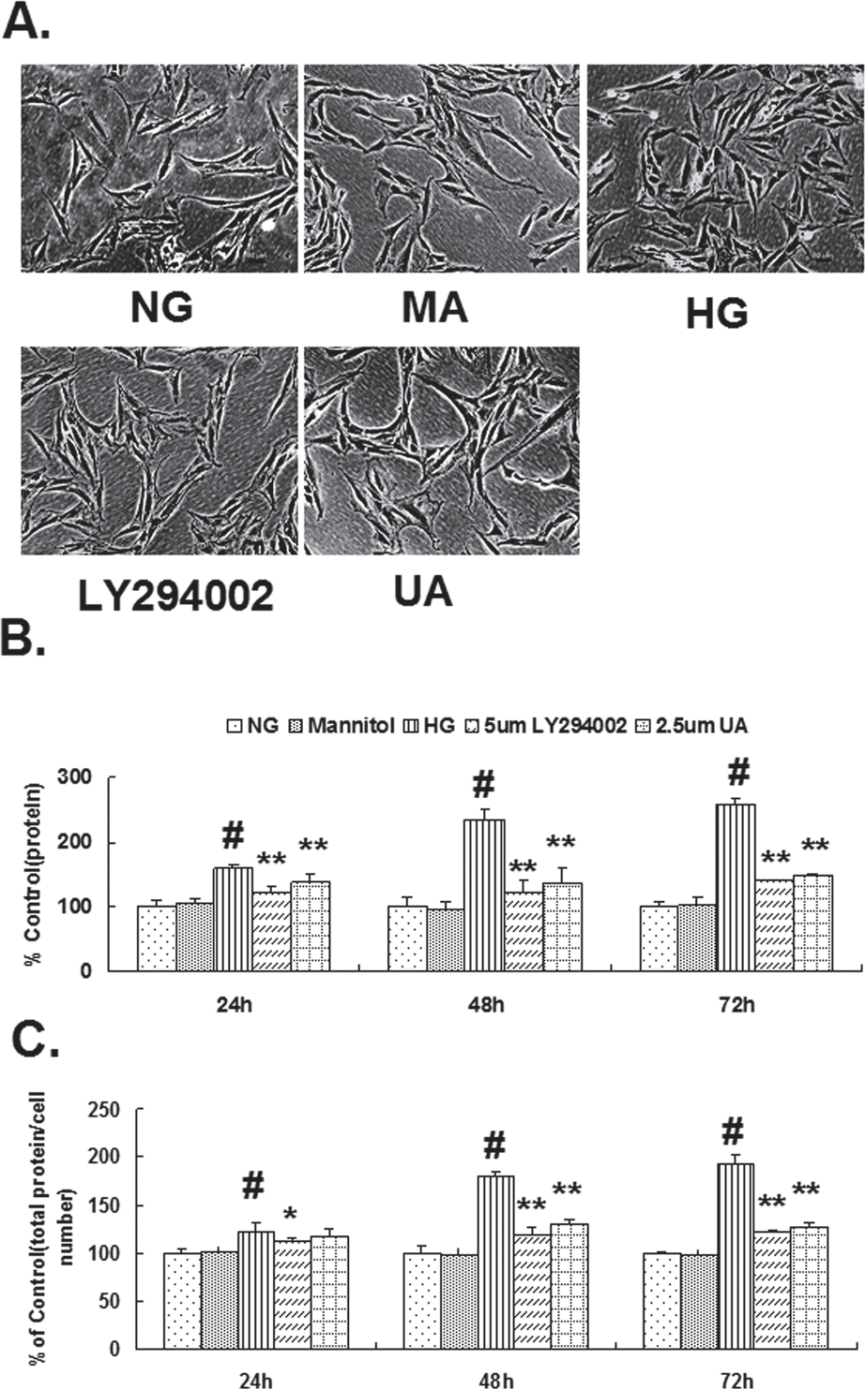
Ursolic acid inhibited HG-induced rat mesangial cell abnormal hypertrophy. (A) Images of cells cultured for 48 h in each individual group through inverted phase contrast microscope (200x), Bar = 50 μm. (B) The total protein content in each group was measured using BCA. (C) The total protein/cell number ratio expressed as μg/10^4^ cells. #, P<0.01 vs. NG; *, P<0.05 vs. HG; **, P<0.01 vs. HG.

(1). Compared with the normal glucose control group, there was no obvious proliferation of the cells cultured under HG conditions for 24 h; however, after 48 h of culture, the proliferation rate significantly increased (P<0.01). Compared with the HG group, the proliferation rates of the cells in the intervention groups treated with UA at concentrations of 5.0 and 7.5 μmol/L were significantly decreased after 24 h (P<0.01). The cell proliferation in the intervention group that received 1.5–2.5 μmol/L UA was significantly decreased after 48 h (P<0.01). The proliferation rate in the LY294002 group was significantly decreased after 24 h (P<0.01). The maximum inhibition ratios for the cells in the intervention groups treated with UA at concentrations of 5.0 and 7.5 μmol/L and the LY294002 group were 26%, 30% and 8%, respectively; But did not reach the half maximal (50%) inhibitory concentration (IC50) level. The proliferation rates between the hypertonic and the normal glucose control group were not significantly different at any time point, indicating that the abnormal cell proliferation induced through HG was not caused by high osmotic pressure (Fig. 1). The cell proliferation rate in the 2.5 μmol/L UA intervention group was significantly lower than that in the HG group, but this rate was not lower than that observed in the normal glucose control group. Therefore, the optimal UA concentration selected for this study was 2.5 μmol/L.

(2). Compared with normal glucose control group, the amount of total protein and the degree of hypertrophy in the hypertonic group were not significantly different. The total protein and degree of hypertrophy in cells cultured under HG conditions for 24 h were both significantly increased (P<0.01). Compared with the HG group, the total protein in the cells in the UA intervention group was significantly decreased after 24 h (P<0.01), whereas the degree of hypertrophy was significantly decreased after 48 h (P<0.01). The total protein and degree of hypertrophy in the LY294002 group were both decreased after 24 h (P<0.05; Fig. 2).

### 2. Down-regulation of the expression of type I collagen in mesangial cells cultured under HG conditions after UA treatment

**Fig 3:**
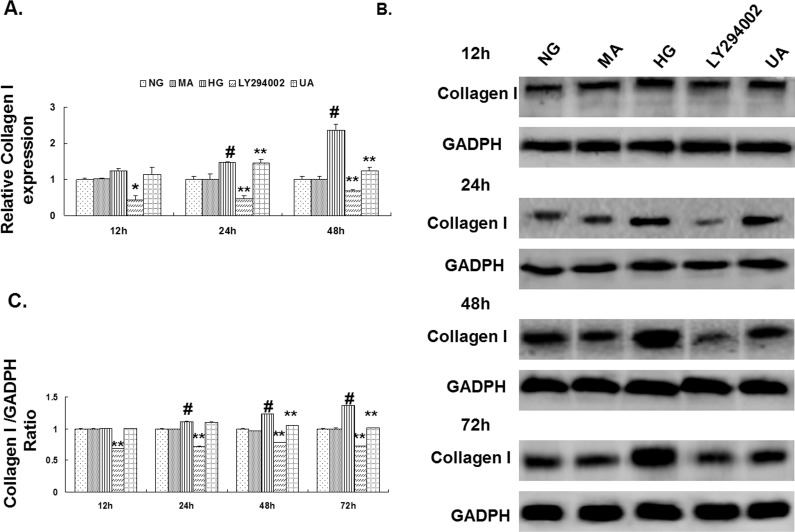
Ursolic acid reduced HG-induced collagen I generation in rat mesangial cells. (A) RT-qPCR analysis of collagen I mRNA expression in each group of cells cultured for 12, 24 and 48 h. (B) Western blot analysis of the collagen I protein expression in each group of cells cultured for 12, 24, 48 and 72 h. (C) The intensities of the bands for collagen I protein in (B) were quantified. #, P<0.01 vs. NG; *, P<0.05 vs. HG; **, P<0.01 vs. HG.

The expression of type I collagen mRNA and protein in mesangial cells was detected using reverse transcriptase quantitative PCR (RT-qPCR) and western blotting. The results showed that the expression of type I collagen mRNA and protein was significantly increased in mesangial cells after culture under HG conditions for 24 h compared with the normal glucose control group (P<0.01). The expression of type I collagen in the cells in the hypertonic group was not significantly different from the expression in the normal glucose control group at any time point. Compared with the HG group, the type I collagen mRNA and protein expression in the UA intervention group was significantly decreased after 24 and 48 h, respectively (P<0.01). The type I collagen expression in the mesangial cells in the LY294002 group was significantly decreased after 12 h of culture (P<0.05; Fig. 3).

### 3. Attenuation of HG-induced inhibition of autophagy in mesangial cells through UA

**Fig 4:**
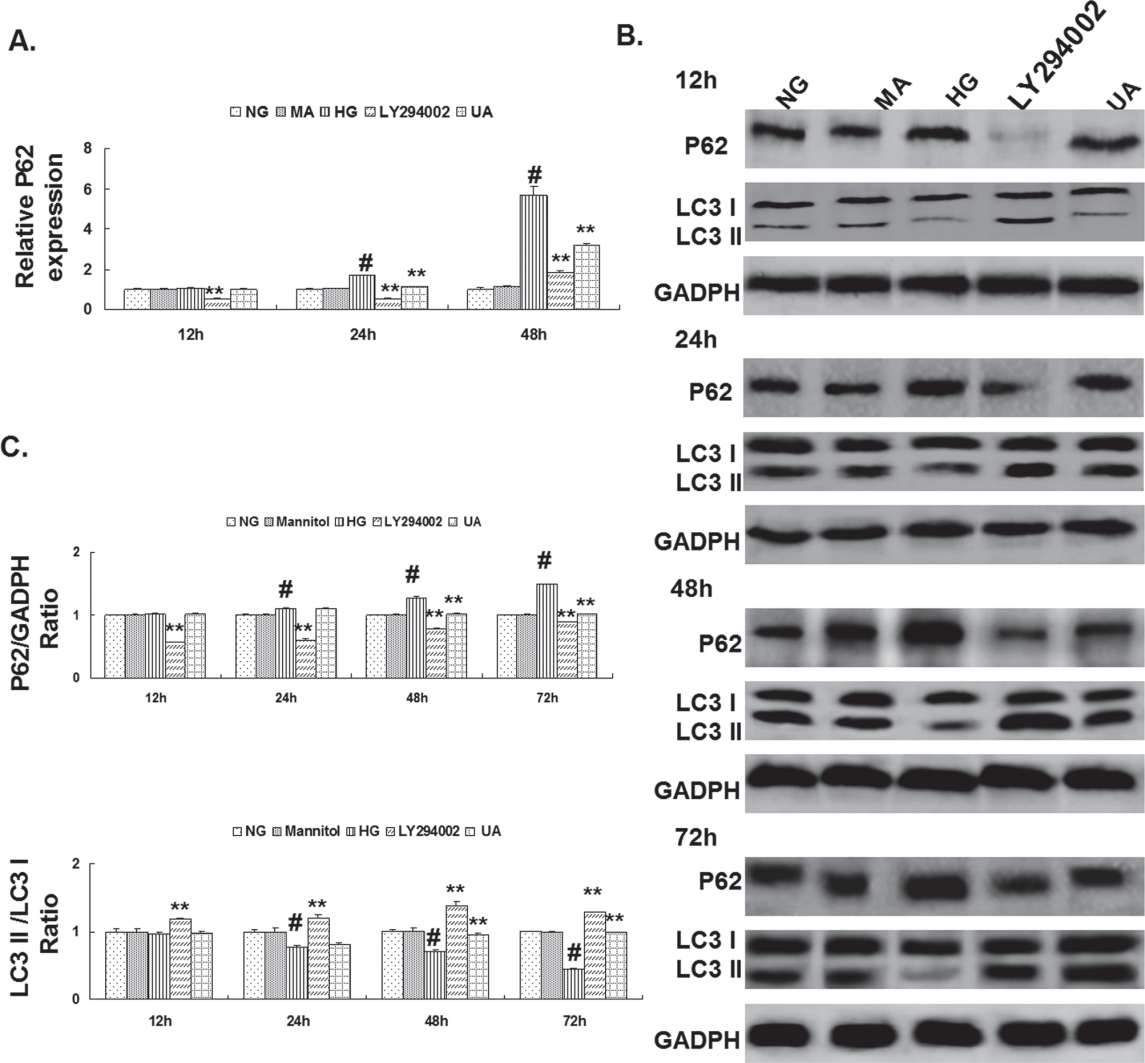
Ursolic acid attenuated HG-induced rat mesangial cell autophagy inhibition. (A) RT-qPCR analysis of p62/SQSTMI mRNA expression in each group of cells cultured for 12, 24 and 48 h. (B) Western blot analysis of LC3II and p62/SQSTMI protein expression in each group of cells cultured for 12, 24, 48 and 72 h. (C) The intensities of bands for LC3II and p62/SQSTMI protein in (B) were quantified. #, P<0.01 vs. NG; *, P<0.05 vs. HG; **, P<0.01 vs. HG.

The expression of p62/sequestosome-1 (SQSTM1) mRNA in mesangial cells was detected using RT-qPCR, and the expression of the p62/SQSTMI and LC3II proteins was detected using western blotting. The results showed that the expression of p62/SQSTMI mRNA and protein in mesangial cells cultured under HG conditions for 24 h was significantly up-regulated compared with the normal glucose control group, whereas the expression of LC3II was significantly down-regulated (P<0.01). The expression of p62/SQSTMI and LC3II in the hypertonic group at different time points was not significantly different compared with the normal glucose control group. Compared with the HG group, the expression of p62/SQSTMI mRNA in mesangial cells in the UA intervention group was significantly down-regulated after 24 h, whereas the expression of p62/SQSTMI protein was significantly down-regulated and LC3II protein was significantly up-regulated after 48 h (P<0.01). After culturing for 12 h, the expression of p62/SQSTMI mRNA and protein in the LY294002 group was significantly down-regulated, whereas the expression of LC3II protein was significantly up-regulated (P<0.01; Fig. 4).

### 4. Transmission electron microscopy reveals the inhibition of HG-induced autophagosome reduction in mesangial cells through UA

**Fig 5:**
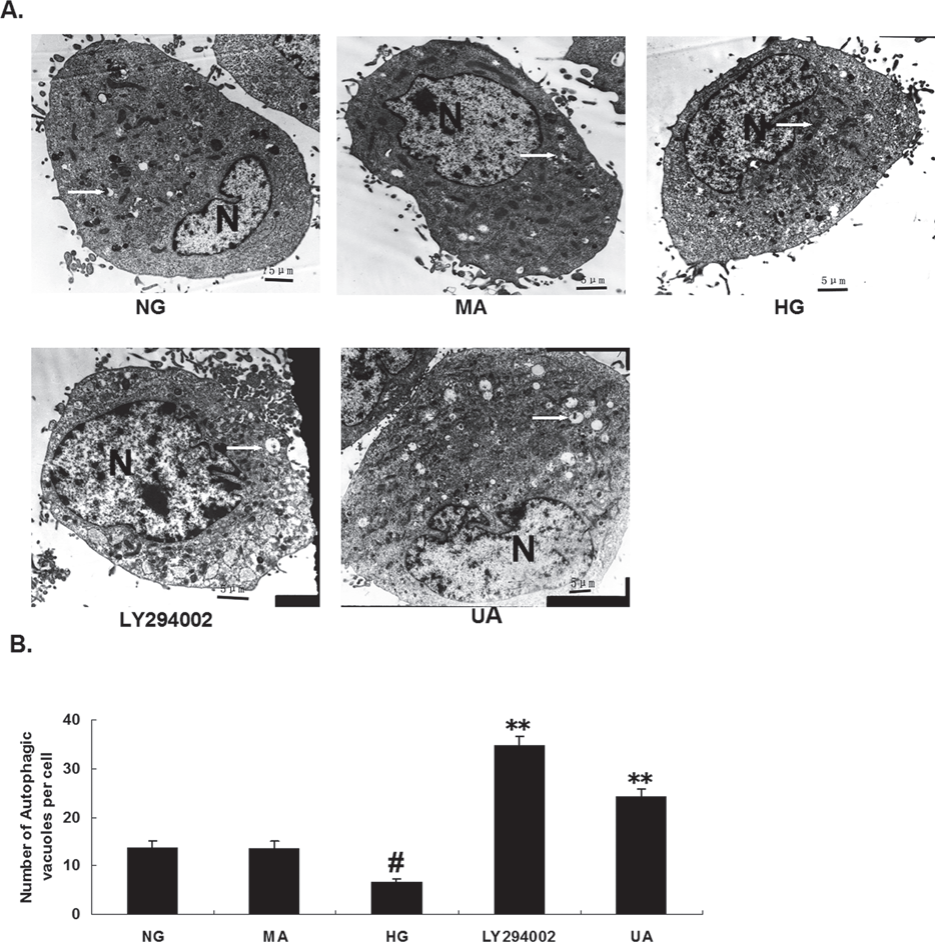
Transmission electron microscopy (4000x) showing that ursolic acid increases the number of autophagic vacuoles in rat mesangial cells. (A) TEM micrograph: the “N” represents nucleus and the arrows indicate autophagosomes. Numerous autophagic vacuoles with typical double-layer membrane structures containing undigested organelle remnants are indicated with arrows. Bar = 5 μm. (B) The number of autophagosomes was counted in 10 randomly selected cells. #, P<0.01 vs. NG; *, P<0.05 vs. HG; **, P<0.01 vs. HG.

After 48 h of culture, the intracellular autophagosome formation in the cells from each group was examined through transmission electron microscopy. The number of autophagosomes in every 10 fields was counted. Compared with the normal glucose control group, the number of autophagosomes in the mesangial cells in the HG group was significantly decreased (P<0.01). The number of autophagosomes in the mesangial cells in the hypertonic group was not significantly different compared with the normal glucose control group. Compared with the HG group, the number of autophagosomes in the UA and LY294002 groups was significantly increased (P<0.01; Fig. 5).

### 5. Inhibition of HG-induced up-regulated miRNA-21 expression, down-regulated PTEN expression, and PI3K/AKT/mTOR signaling pathway activation through UA

**Fig 6:**
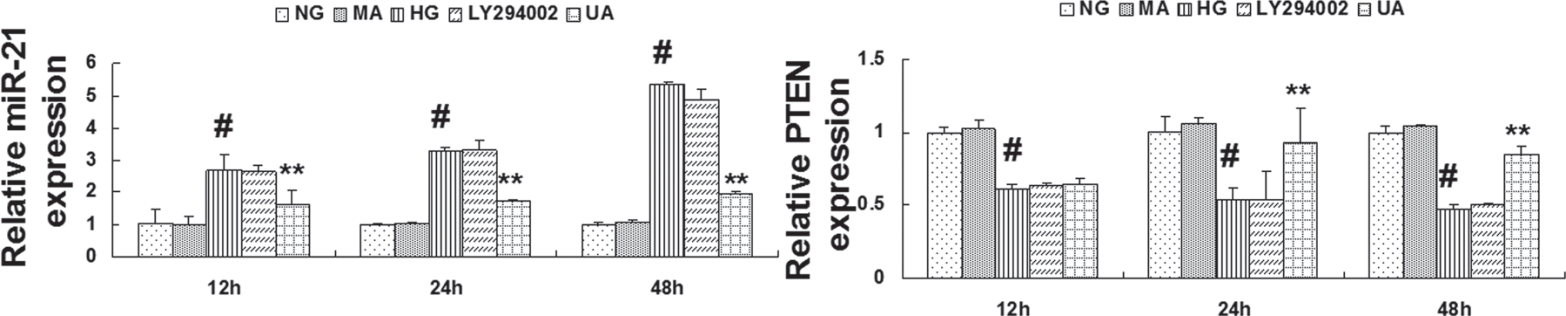
Ursolic acid inhibited the HG-induced up-regulation of miRNA-21 mRNA expression, down-regulation of PTEN mRNA expression. RT-qPCR analysis for miRNA-21 and PTEN mRNA expression in each group of cells cultured for 12, 24 and 48 h. #, P<0.01 vs. NG; *, P<0.05 vs. HG; **, P<0.01 vs. HG.

**Fig 7:**
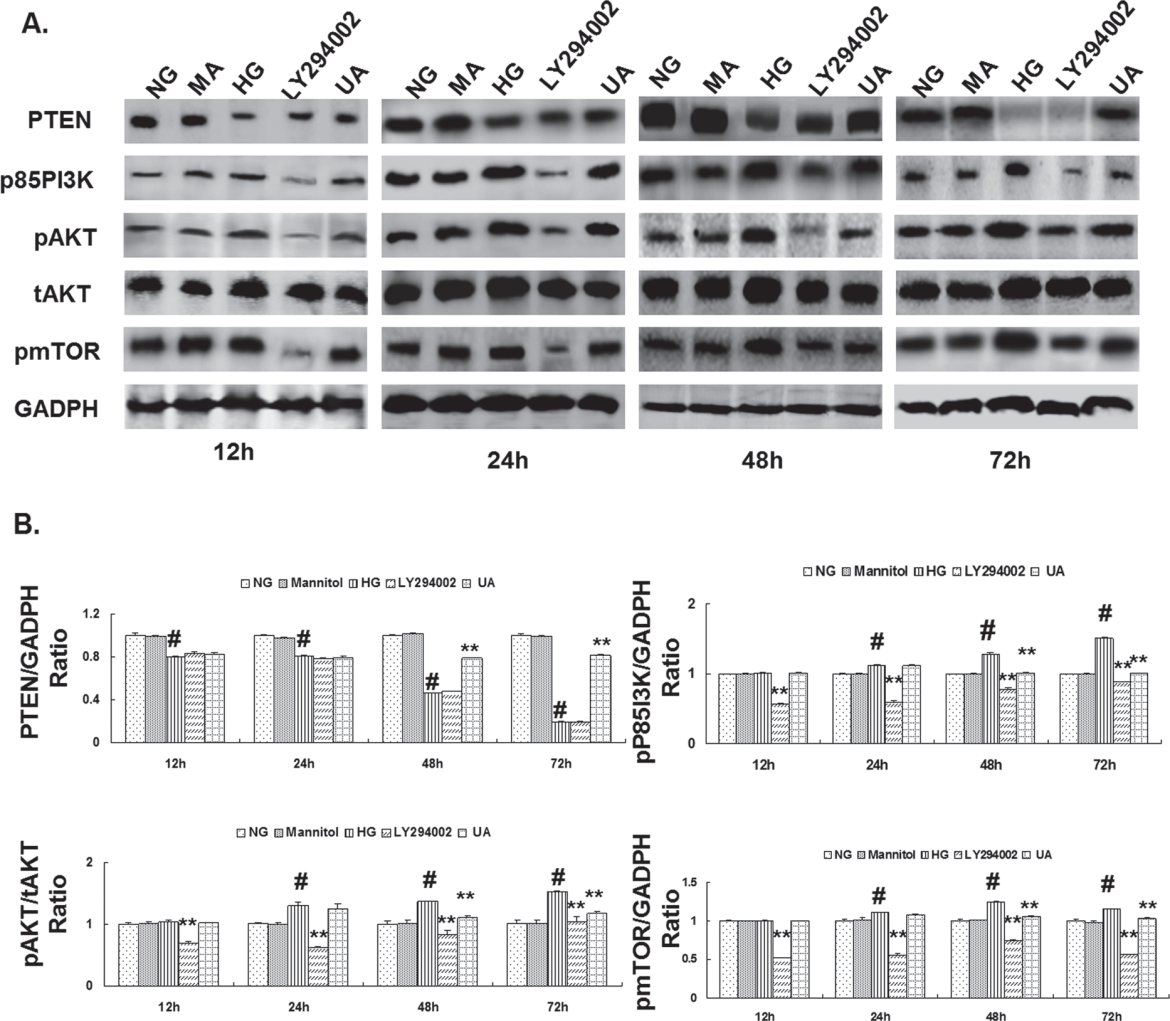
Ursolic acid inhibited the HG-induced down-regulation of PTEN expression, and activation of the PI3K/AKT/mTOR signaling pathway in rat mesangial cells. (A) Western blot analysis for PTEN, p85-PI3K, pAKT, tAKT, and pmTOR protein expression in each group of cells cultured for 12, 24, 48 and 72 h. (B) The intensities of the bands for PTEN, p85-PI3K, pAKT, and pmTOR protein in (A) were quantified. #, P<0.01 vs. NG; *, P<0.05 vs. HG; **, P<0.01 vs. HG.

1. Compared with the normal glucose control group, miRNA-21 expression was significantly up-regulated, and PTEN mRNA expression was significantly down-regulated in mesangial cells cultured under HG conditions for 12 h (P<0.01). The expression of miRNA-21 and PTEN mRNA in the hypertonic group at different time points was not significantly different compared with the normal glucose control group. Compared with the HG group, miRNA-21 expression was significantly down-regulated in the UA intervention group after 12 h, whereas PTEN mRNA expression was significantly up-regulated after 24 h (P<0.01). The expression of miRNA-21 and PTEN mRNA in the LY294002 group at different time points was not significantly different compared with the HG group (Fig. 6).

2. The expression of PTEN, p85 PI3K, pAKT, tAKT, and pmTOR was detected using western blotting. The results indicated that the PTEN expression in the mesangial cells cultured under HG conditions was significantly down-regulated after 12 h, whereas the expression of p85 PI3K, pAKT, and pmTOR was significantly up-regulated after 24 h (P<0.01). The expression of PTEN, p85 PI3K, pAKT, and pmTOR in the hypertonic group at different time points was not significantly different from the normal glucose control group. Compared with the HG group, PTEN expression was significantly up-regulated in the UA intervention group after 48 h, whereas the expression of p85 PI3K, pAKT, pmTOR was significantly down-regulated (P<0.01). After culture for 12 h, the expression of p85 PI3K, pAKT, tAKT, and pmTOR in the mesangial cells in the LY294002 group was significantly decreased (P<0.01), whereas the expression of PTEN at different time points was not significantly different compared with the HG group (Fig. 7).

## Discussion

Recent studies have indicated that UA inhibits tumor cell growth through the inhibition of the Akt/mTOR signaling pathway [18–20]. UA also improves streptozotocin (STZ)-induced glomerular hypertrophy and the increased extracellular matrix in DN rats through inhibition of the activation of the signal transducer and activator of transcription (STAT) 3, extracellular signal-regulated kinases (ERK)1/2, and c-Jun N-terminal kinase (JNK) pathways [21]. However, the specific renal protective mechanisms of UA remain unclear. Currently, it remains unknown whether UA exerts renal protection effects through the inhibition of miRNA-21 expression in mesangial cells.

Renal enlargement, one of the first structural changes in DN, reflects the hypertrophy of existing glomerular and tubular cells rather than cellular proliferation [22–25]. A large number of studies have confirmed that the activation of the Akt/mTOR signaling pathway plays an important role in DN pathogenesis [26–30]. The activation of the Akt/mTOR signaling pathway leads to cell hypertrophy, the accumulation of extracellular matrix, and resident renal cell injury. Previous *in vivo* studies have shown that decreased PTEN expression and PI3K/Akt pathway activation promote DN occurrence and development [31]. The results of the present study confirmed that PTEN expression was significantly decreased in mesangial cells cultured under HG conditions after 12 h. We also demonstrated that the abnormal activation of the PI3K/Akt/mTOR signaling pathway and abnormal cell hypertrophy occurred after 24 h, and abnormal cell proliferation was observed after 48 h. The results of the present study also confirmed that cell hypertrophy occurs before cell proliferation. Indeed, the results of the present study showed that PTEN expression was significantly up-regulated at 48 h after UA treatment. In addition, the activation of the PI3K/Akt/mTOR signaling pathway was also inhibited, and mesangial cell hypertrophy and proliferation was improved at 48 h after UA treatment. After 24 h of LY294002 treatment, the activation of the PI3K/Akt/mTOR signaling pathway was significantly inhibited, but PTEN expression was not affected. These results indicate that UA might inhibit the activation of PI3K and the Akt/mTOR signaling pathway through PTEN up-regulation. Future studies should use PTEN inhibitors to determine whether UA inhibits the activation of the Akt/mTOR signaling pathway through PTEN up-regulation.

Current studies have shown that the PI3K/Akt/mTOR signaling pathway is abnormally activated in the kidneys of diabetes mellitus patients; however, the specific mechanism underlying this activation remains unclear. Increasing evidence indicates that miRNA plays important roles in the activation of the PI3K/Akt/mTOR signaling pathway. Current studies on diabetic processes indicate that miRNA-216, miRNA-217, and miRNA-21 target PTEN [6], and the miRNA-200 family targets friend of GATA (FOG)2 [32] to activate the Akt/mTOR signaling pathway, thereby mediating DN occurrence and development. Dey et al. [33,34] showed that HG and transforming growth factor (TGF)-β increase miRNA-21 expression, while miRNA-21 targets the 3'-untranslated region (UTR) of PTEN mRNA to inhibit PTEN protein expression. The over-expression of miRNA-21 decreases PTEN expression and simultaneously activates the Akt/mTOR signaling pathway, thereby inducing mesangial cell hypertrophy and mesangial matrix expansion. In contrast, the inhibition of endogenous miRNA-21 expression blocks HG-induced PTEN down-regulation and Akt phosphorylation. These results suggest that miRNA-21 participates in the occurrence of key pathological injuries in DN through a reduction in the level of the target protein PTEN and the activation of the Akt/mTOR pathway. The results of the present study showed simultaneous miRNA-21 up-regulation and PTEN down-regulation in mesangial cells after 12 h of culture under HG conditions, thus activating the Akt/mTOR pathway. UA treatment significantly inhibited HG-induced miRNA-21 up-regulation after 12 h and up-regulated PTEN mRNA expression after 24 h, resulting in up-regulated PTEN protein expression and the inhibition of the Akt/mTOR pathway after 48 h. Treatment with LY294002 also induced miRNA-21 up-regulation and the down-regulation of PTEN expression after 12 h, and these changes were not significantly different compared with the HG group. These results indicate that HG up-regulated miRNA-21 and PTEN expression to activate the PI3K/Akt/mTOR pathway. In addition, UA up-regulated PTEN mRNA and protein expression through the inhibition of miRNA-21 up-regulation, thus inhibiting the abnormal activation of the Akt/mTOR pathway and attenuating mesangial cell injury induced through HG.

Autophagy [35] is tightly regulated to ensure an optimal balance between synthesis and degradation and the use, storage and recycling of cellular products. Autophagy also plays an important role in the maintenance of renal cell function. The balance between the synthesis and degradation of extracellular matrix (ECM) [36] proteins is crucial to maintain tissue homeostasis. The continuous production and progressive accumulation of the ECM hallmarks renal fibrosis in DN. Collagens are the primary components of the ECM in the kidney, and type I collagen, produced from mesangial cells, is primarily associated with disease states [37–39]. Recent evidence suggests an additional mechanism by which I collagen, a major ECM component, undergoes intracellular degradation through the induction of autophagy [40,41]. Recent studies have indicated that the deregulation of autophagy and the pathogenesis of DN are closely associated. When autophagy is down-regulated under energy-rich conditions, the long-term inhibition of autophagy results in organelle injury and the accumulation of products destined for degradation [42]. Previous studies have shown a reduction in autophagy functions in animal models of diabetes mellitus and in mesangial cells cultured under HG conditions. When injured or senescent organelles (such as mitochondria) or macromolecules (such as proteins) in cells cannot be efficiently cleared, large amounts of these substances accumulate inside cells, aggravating cell injury and promoting senescence [43,44]. Kim et al. showed that autophagy degraded excess type I collagen and decreased the accumulation of extracellular matrix in kidney cells, thus attenuating renal fibrosis and protecting these cells [45]. A recent study showed that UA induces autophagy in cervical cancer cells, thereby inhibiting cancer cell growth [46]. The results of the present study showed that the expression of type I collagen and p62/SQSTMI was increased and the expression of LC3II was decreased in mesangial cells after 24 h of culture under HG conditions, indicating that the production of type I collagen increased with decreasing autophagy function. Treatment with UA decreased the expression of type I collagen and p62/SQSTMI mRNA after 24 h, whereas the expression of type I collagen and p62/SQSTMI protein was decreased and the expression of LC3II protein was increased after 48 h. These results indicate that UA significantly enhances autophagy functions in cells and simultaneously decreases the accumulation of type I collagen. Future studies will investigate whether UA directly affects the expression of autophagy-related genes or specific miRNAs, such as miR-196, miR-101, and miR30A, which in turn regulate the expression of autophagy-related genes.

**Fig 8:**
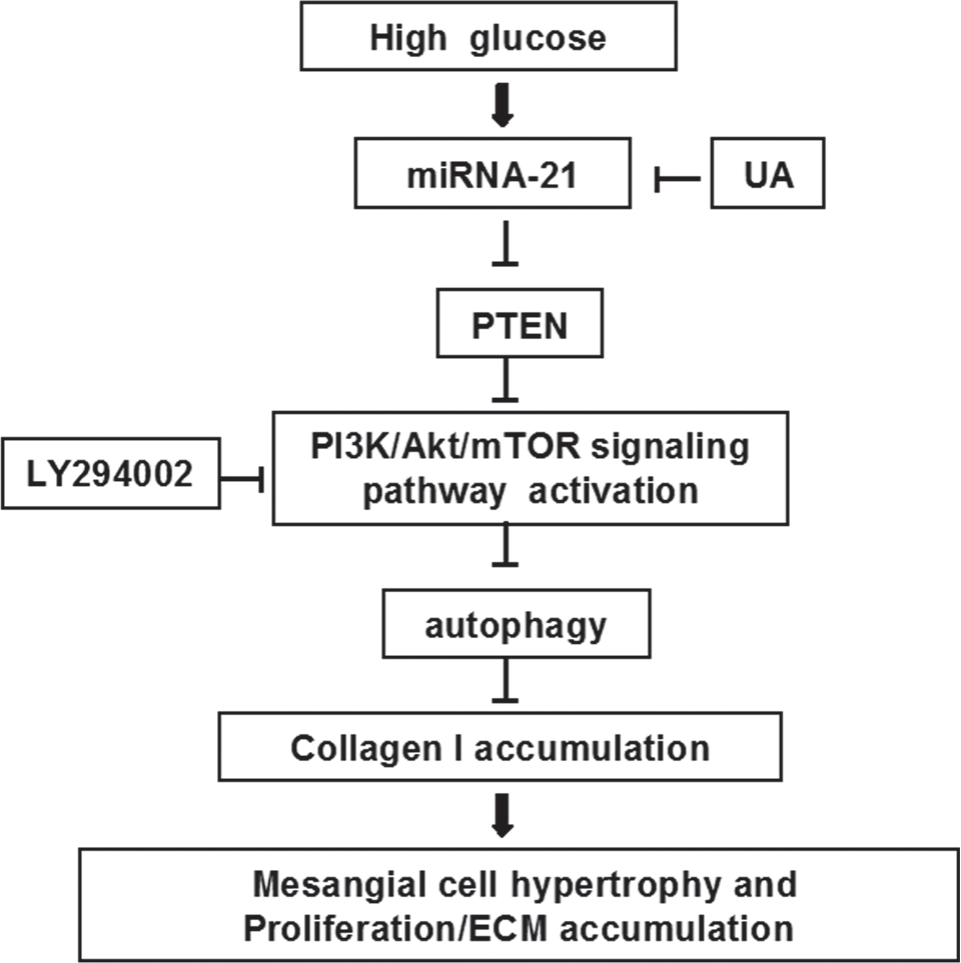
Ursolic acid attenuates HG-induced mesangial cell injury through the up-regulation of autophagy, at least in part, through the suppression of the miR21/PTEN/Akt/mTOR pathway. HG stimulates the up-regulation of miR21 expression, resulting in the down-regulation of PTEN expression and the activation of PI3K/Akt/mTOR. Ursolic acid might inhibit HG-induced cell proliferation and hypertrophy through the inhibition of this pathway.

In summary, the results of the present study demonstrated that the up-regulation miRNA-21 expression, down-regulation of PTEN expression, and abnormal activation of the PI3K/Akt/mTOR signaling pathway occur in mesangial cells cultured under HG conditions, thereby decreasing autophagy and increasing the accumulation of type I collagen in mesangial cells, resulting in abnormal hypertrophy and cell proliferation. Through the inhibition of miRNA-21 expression, UA treatment up-regulated PTEN expression, inhibited PI3K/Akt/mTOR signaling, attenuated mesangial autophagy inhibition under diabetic conditions, reduced abnormal hypertrophy and mesangial cell proliferation, reduced type I collagen accumulation, and exerted a renal protective function (Fig. 8). The results of the study from other members of our research group have indicated that UA Inhibits mesangial cell proliferation and induces apoptosis through the inhibition of HG-induced up-regulated anti-apoptotic factor Bcl-xl expression, down-regulated pro-apoptotic factor Bax expression and AKT/mTOR signaling pathway activation. These results indicate UA inhibits high glucose-induced mesangial cell proliferation, probably through the combination of inducing autophagy and apoptosis. Thus, UA might represent a valuable therapeutic drug to block the progression of DN, specifically targeting PI3K/AKT/mTOR activation. Next, we will perform further *in vivo* experiments, transfecting miRNA-21 over-expression plasmids and knockout plasmids to verify the mechanisms underlying the renal protective functions of UA.

## References

[1] Ravikumar B, Starker S, Davies JE, Futter M, Garcia-Arencibia M, Green-Thompson ZW, et al (2010) Regulation of mammalian autophagy in physiology and pathophysiology. Physiol Rev 90(4): 1383–1435. 10.1152/physrev.00030.2009 20959619

[2] Komatsu M, Waguri S, Koike M, Sou YS, Ueno T, et al (2007) Homeostatic levels of p62 control cytoplasmic inclusion body formation in autophagy-deficient mice.Cell 131(6):1149–63. 18083104 10.1016/j.cell.2007.10.035

[3] Bjørkøy G, Lamark T, Brech A, Outzen H, Perander M, et al (2005) p62/SQSTM1 forms protein aggregates degraded by autophagy and has a protective effect on huntingtin-induced cell death. J Cell Biol 171(4):603–14. 16286508 10.1083/jcb.200507002 PMC2171557

[4] Hartleben B, Gödel M, Meyer-Schwesinger C, Liu S, Ulrich T, et al (2010) Autophagy influences glomerular disease susceptibility and maintains podocyte homeostasis in aging mice. J Clin Invest 120(4):1084–1096. 10.1172/JCI39492 20200449 PMC2846040

[5] García Z, Kumar A, Marqués M, Cortés I, Carrera AC (2006) Phosphoinositide 3-kinase controls early and late events in mammalian cell division.EMBO J 25(4), 655–661. 16437156 10.1038/sj.emboj.7600967 PMC1383550

[6] Chandrasekaran K, Karolina DS, Sepramaniam S, Armugam A, Wintour EM, et al (2012) Role of microRNAs in kidney homeostasis and disease. Kidney Int 81(7):617–627. 10.1038/ki.2011.448 22237749

[7] Kasinath BS, Feliers D (2011) The complex world of kidney microRNAs. Kidney Int. 80(4):334–7. 10.1038/ki.2011.165 21799505

[8] Ikeda Y, Murakami A, Ohigashi H (2008) Ursolic acid: An anti- and pro-inflammatory triterpenoid. Mol.Nutr. Food Res 52(1):26–42. 10.1002/mnfr.200700389 18203131

[9] Somova LO, Nadar A, Rammanan P, Shode FO (2003) Cardiovascular, antihyperlipidemic and antioxidant effects of oleanolic and ursolic acids in experimental hypertension. Phytomedicine 10(2–3):115–121. 12725563 10.1078/094471103321659807

[10] Jayaprakasam B, Olson LK, Schutzki RE, Tai MH, Nair MG (2006) Amelioration of obesity and glucose intolerance in high-fat-fed C57BL/6 mice by anthocyanins and ursolic acid in Cornelian cherry(Cornus mas). J Agric Food Chem 54(1):243–248. 16390206 10.1021/jf0520342

[11] Liobikas J, Majiene D, Trumbec kaite S, Kursvie tiene L, Masteikova R, et al (2011) Uncoupling and antioxidant effects of ursolic acid in isolated rat heart mitochondria. J N at Prod 74(7): 1640–1644. 10.1021/np200060p 21648406

[12] Kunkel SD, Elmore CJ, Bongers KS, Ebert SM, Fox DK, et al (2012) Ursolic acid increases skeletal muscle and brown fat and decreases diet- induced obesity, glucose intolerance and fatty liver disease. PLoS One 7(6): e39332 10.1371/journal.pone.0039332 22745735 PMC3379974

[13] Lee J, Yee ST, Kim JJ, Choi MS, Kwon EY, et al (2010) Ursolic acid ameliorates thymic atrophy and hyperglycemia in streptozotocin-nicotinamide-induced diabetic mice. Chem Biol Interact 188(3): 635–642. 10.1016/j.cbi.2010.09.019 20869956

[14] Leng S, Hao Y, Du D, Xie S, Hong L, et al (2013) Ursolic acid promotes cancer cell death by inducing Atg5-dependent autophagy. Int J Cancer 133(12): 2781–2790. 10.1002/ijc.28301 23737395

[15] Wang X, Ikejima K, Kon K, Arai K, Aoyama T, et al (2011) Ursolic acid ameliorates hepatic fibrosis in the rat by specific induction of apoptosis in hepatic stellate cells. J Hepato 55(2):379–87. 10.1016/j.jhep.2010.10.040 21168456

[16] Pai PG, Chamari Nawarathna S, Kulkarni A, Habeeba U, Reddy CS, et al (2012) Nephroprotective effect of ursolic Acid in a murine model of gentamicin-induced renal damage. ISRN Pharmacol 2012:410902 10.5402/2012/410902 22811930 PMC3394390

[17] Wang J, Li Y, Wang X, Jiang C (2012) Ursolic acid inhibits proliferation and induces apoptosis in human glioblastoma cell lines U251 by suppressing TGF-β1/miR-21/PDCD4 pathway. Basic Clin Pharmacol Toxicol. 111(2):106–112. 10.1111/j.1742-7843.2012.00870.x 22353043

[18] Achiwa Y, Hasegawa K, Udagawa Y (2007) Regulation of the Phosphatidylinositol 3-Kinase-AKT and the Mitogen-Activated Protein Kinase Pathways by Ursolic Acid in Human Endometrial Cancer Cells. Biosci Biotechnol Biochem 71(1):31–37. 17213663 10.1271/bbb.60288

[19] Shin SW, Kim SY, Park JW (2012) Autophagy inhibition enhances ursolic acid-induced apoptosis in PC3 cells. Biochim Biophys Acta 1823(2):451–457. 10.1016/j.bbamcr.2011.10.014 22178132

[20] Wu B, Wang X, Chi ZF, Hu R, Zhang R, et al (2012) Ursolic acid-induced apoptosis in K562 cells involving upregulation of PTEN gene expression and inactivation of the PI3K/Akt pathway. Arch Pharm Res 35(3):543–8. 10.1007/s12272-012-0318-1 22477202

[21] Zhou Y, Li JS, Zhang X, Wu YJ, Huang K, et al (2010) Ursolic acid inhibits early lesions of diabetic nephropathy. Int J Mol Med 26(4):565–570. 20818497 10.3892/ijmm_00000500

[22] Estacio RO, Schrier RW (2001) Diabetic nephropathy: Pathogenesis, diagnosis, and prevention of progression. Adv Intern Med 46:359–408. 11147259

[23] Molitch ME, DeFronzo RA, Franz MJ, Keane WF, Mogensen CE, et al (2004) Nephropathy in diabetes. Diabetes Care 27[Suppl 1]: S79—S83. 14693934 10.2337/diacare.27.2007.s79

[24] Hostetter TH (1995) Progression of renal disease and renal hypertrophy. Annu Rev Physiol 57: 263–278. 7778868 10.1146/annurev.ph.57.030195.001403

[25] Hostetter TH (2003) Hyperfiltration and glomerulosclerosis. Semin Nephrol 23(2): 194–199. 12704579 10.1053/anep.2003.50017

[26] Mavroeidi V, Petrakis I, Stylianou K, Katsarou T, Giannakakis K, et al (2013) Losartan Affects Glomerular AKT and mTOR Phosphorylation in an Experimental Model of Type 1 Diabetic Nephropathy. J Histochem Cytochem 61(6):433–443. 10.1369/0022155413482925 23456824 PMC3715326

[27] Hafizi S, Wang X, Chester AH, Yacoub MH, Proud CG (2004) ANG II activates effectors of mTOR via PI3K signaling in human coronary smooth muscle cells. Am J Physiol Heart Circ Physiol 287(3):H1232–1238. 15317677 10.1152/ajpheart.00040.2004

[28] Hers I, Vincent EE, Tavaré JM (2011) Akt signalling in health and disease. Cell Signal 23(10):1515–1527. 10.1016/j.cellsig.2011.05.004 21620960

[29] Sekulić A, Hudson CC, Homme JL, Yin P, Otterness DM, et al (2000) A direct linkage between the phosphoinositide 3-kinase-AKT signaling pathway and the mammalian target of rapamycin in mitogen-stimulated and transformed cells. Cancer Res 60(13):3504–3513. 10910062

[30] Lieberthal W, Levine JS (2009) The role of the mammalian target of rapamycin (mTOR) in renal disease. J Am Soc Nephrol 20(12): 2493–2502. 10.1681/ASN.2008111186 19875810

[31] Wang YY, Liu RX, Guo B, Xiao Y, Shi MJ, et al (2011) Down-regulation of PTEN expression in kidney and its role in development of diabetic nephropathy in rats. Sheng Li Xue Bao 63(4):325–332. 21861051

[32] Hyun S, Lee JH, Jin H, Nam J, Namkoong B, et al (2009) Conserved MicroRNA miR-8/miR-200 and its target USH/FOG2 control growth by regulating PI3K. Cell 139(6):1096–1108. 10.1016/j.cell.2009.11.020 20005803

[33] Dey N, Ghosh-Choudhury N, Kasinath BS, Choudhury GG (2012) TGFβ-Stimulated MicroRNA-21 Utilizes PTEN to Orchestrate AKT/mTORC1 Signaling for Mesangial Cell Hypertrophy and Matrix Expansion. PLoS One 7(8):e42316 10.1371/journal.pone.0042316 22879939 PMC3411779

[34] Dey N, Das F, Mariappan MM, Mandal CC, GhoshChoudhury N, et al (2011) MicroRNA-21 orchestrates high glucose-induced signals to TOR complex 1, resulting in renal cell pathology in diabetes. J Biol Chem 286(29):25586–25603. 10.1074/jbc.M110.208066 21613227 PMC3138272

[35] Mizushima N (2009) Physiological functions of autophagy. Curr Top Microbiol Immunoln 335:71–84. 10.1007/978-3-642-00302-8_3 19802560

[36] Vu TH, Werb Z (2000) Matrix metalloproteinases: effectors of Development and normal physiology.Genes Dev 14(17), 2123–2133. 10970876 10.1101/gad.815400

[37] Yoshioka K, Tohda M, Takemura T, Akano N, Matsubara K, et al (1990) Distribution of type I collagen in human kidney diseases in comparison with type III collagen. J. Pathol 162(2):141–148. 2250192 10.1002/path.1711620207

[38] Chin BY, Mohsenin A, Li SX, Choi AM, Choi ME (2001) Stimulation of pro- (1)(I) collagen by TGF-(1) in mesangial cells: role of the p38 MAPK pathway. Am. J. Physiol Renal Physiol 280(3): F495–F504. 11181412 10.1152/ajprenal.2001.280.3.F495

[39] Kim SI, Kwak JH, Zachariah M, He Y, Wang L, et al (2007) TGF-beta-activated kinase 1 and TAK1-binding protein 1 cooperate to mediate TGF-1-induced MKK3-p38 MAPK activation and stimulation of type I collagen. Am J Physiol Renal Physiol 292(5):F1471–F1478. 17299140 10.1152/ajprenal.00485.2006

[40] Ishida Y, Yamamoto A, Kitamura A, Lamandé SR, Yoshimori T, et al (2009) Autophagic elimination of misfolded procollagen aggregates in the endoplasmic reticulum as a means of cell protection.Mol Biol Cell 20(11):2744–2754. 10.1091/mbc.E08-11-1092 19357194 PMC2688553

[41] Aránguiz-Urroz P, Canales J, Copaja M, Troncoso R, Vicencio JM, et al (2011) Beta(2)-adrenergic receptor regulates cardiac fibroblast autophagy and collagen degradation. Biochim Biophys Acta 1812(1):23–31. 10.1016/j.bbadis.2010.07.003 20637865

[42] Kume S, Uzu T, Horiike K, Chin-Kanasaki M, Isshiki K, et al (2010) Calorie restriction enhances cell adaptation to hypoxia through Sirt1-dependent mitochondrial autophagy in mouse aged kidney. J Clin Invest 120(4):1043–1055. 10.1172/JCI41376 20335657 PMC2846062

[43] Kitada M, Takeda A, Nagai T, Ito H, Kanasaki K, et al (2011) Dietary restriction ameliorates diabetic nephropathy through anti-inflammatory effects and regulation of the autophagy via restoration of Sirt 1 in diabetic Wistar fatty (fa/fa) rats:a model od type 2 diabetes. Exp Diabetes Res 2011:908185 10.1155/2011/908185 21949662 PMC3178150

[44] Li J, Bai X, Cui S, Fu B, Chen X (2012) Effect of rapamycin on high glucose-induced autophagy impairment, oxidative stress and premature senescence in rat mesangial cells in vitro. Nan Fang Yi Ke Da Xue Xue Bao 32(4):467–471. 22543123

[45] Kim SI, Na HJ, Ding Y, Wang Z, Lee SJ, http://www.ncbi.nlm.nih.gov/pubmed?term=Choi%20ME%5bAuthor%5d&cauthor=true&cauthor_uid=22351764 et al (2012) Autophagy promotes intracellular degradation of type I collagen induced by transforming growth factor (TGF)-β1. J Biol Chem 287(15):11677–11688. 10.1074/jbc.M111.308460 22351764 PMC3320917

[46] Leng S, Hao Y, Du D, Xie S, Hong L, et al (2013) Ursolic acid promotes cancer cell death by inducing Atg5-dependent autophagy. Int J Cancer 133(12):2781–2790. 10.1002/ijc.28301 23737395

